# Corrosion Acceleration of Printed Circuit Boards With an Immersion Silver Layer Exposed to *Bacillus cereus* in an Aerobic Medium

**DOI:** 10.3389/fmicb.2019.01493

**Published:** 2019-07-02

**Authors:** Yuting Hu, Kui Xiao, Dawei Zhang, Pan Yi, Ruilin Xiong, Chaofang Dong, Jusheng Wu, Xiaogang Li

**Affiliations:** Corrosion and Protection Center, University of Science and Technology Beijing, Beijing, China

**Keywords:** PCB-ImAg, corrosion acceleration, *Bacillus cereus*, aerobic medium, growth curve, cross-sectional view

## Abstract

In this research, the corrosion behavior of printed circuit boards with an immersion silver layer (PCB-ImAg) exposed to *Bacillus cereus* bacteria in Luria-Bertani broth was investigated. The growth test demonstrated that *B. cereus* had a high copper tolerance. Analysis of surface and cross-sectional view of the samples after immersion test indicated that metabolites produced by *B. cereus* accelerated the microporous corrosion of PCB-ImAg, and the biofilm that adhered to the surface led to oxygen concentration corrosion. Electrochemical impedance spectroscopy tests confirmed that the microbiologically influenced corrosion of PCB-ImAg was related to the biofilm formation and metabolism.

## Introduction

Microorganisms exist widely in the ocean, soil, and many other environmental systems and grow rapidly when conditions are appropriate (Gu et al., 2019). The surfaces of metallic materials exposed to these environments may rapidly become colonized with microorganisms, resulting in complicated corrosion problems known as microbiologically influenced corrosion (MIC) (Xu and Gu, 2014; Xu et al., 2016; Li et al., 2018). MIC is estimated to account for ∼20% of all corrosion losses, which amount to over 60 billion US dollars each year in China alone (Flemming, 1996; Hou et al., 2017; Huang et al., 2018).

*Bacillus cereus* is a species of bacillus that can enter dormancy by forming spores and withstand adverse conditions, such as high temperature, ultraviolet radiation, and lack of nutrients, and is therefore highly tenacious in poor environments (Nakamura and Silver, 1994; Nourbakhsh et al., 2002; Siddiqui et al., 2005; Shivaji et al., 2006). Novikova et al. (2006) surveyed environmental biocontamination on the International Space Station and isolated a large proportion of *Bacillus* sp. Recently, the impact of this species on materials in various environmental systems has attracted increasing attention. For example, Juzeliūnas et al. (2005) isolated *Bacillus mycoides* from Al, Cu, Zn, and steel samples and investigated accelerated MIC in Zn and inhibition in Al. Qu et al. (2015) reported on the corrosion behavior of cold-rolled steel in artificial seawater containing *Bacillus subtilis*. The strains formed complete biofilms on the surfaces of the samples, which caused the open circuit potential to continuously decrease, and the pH of the broth decreased significantly. MIC by *Bacillus megaterium* has also been found to accelerate the corrosion process of Al-Cu alloys and damage their mechanical properties (Yousaf et al., 2015). Xu et al. (2013) reported that *Bacillus licheniformis* grown as nitrate reducing bacteria, caused a 14.5 μm maximum pit depth on C1018 carbon steel because nitrate reduction coupled with iron oxidation is thermodynamically favorable. The bacteria formed a biofilm around the inclusions and caused damage through metabolic activity (Jia et al., 2019).

With the development of the economy, printed circuit boards (PCBs) are also becoming increasingly miniaturized and complicated, causing PCBs to become very sensitive to factors such as humidity and pollutants in the environment. Copper is widely used in electronic components and tends to corrode. PCB-Cu gradually forms soluble CuCl^2−^ in a Cl^–^-enriched environment, which increases the area of electrolyte layer and accelerates corrosion (Huang et al., 2011). Yi et al. (2016) found that MIC occurred on PCB-Cu exposed in Xishuang Banna. Because there are few pollutants in the local air in Xishuang Banna, the corrosion caused by pollutants and dust is minimal. However, mold spores were found in discolored areas on the samples, and some spores were budding. Immersion silver surface finishing of PCB-Cu is a commonly used method to protect materials from corrosion. Yan et al. (2016, 2017) reported that silver-coated PCB-ImAg had strong resistance to Cl^–^. However, the silver layer is usually thin owing to high cost, which means that there are defects such as micropores on the surface of the layer (Xiao et al., 2017). The copper base and silver layer can form galvanic pairs when PCB-ImAg is exposed to the atmosphere, which causes the copper base to corrode at micropores (Russo et al., 2002). Moreover, silver is sensitive to pollutants containing S, such as H_2_S and SO_2_, and dozens of parts per million of these gasses can cause PCB failure; such concentrations are far below the pollution gas limits required for human health. In environments containing H_2_S, the growth rate of Ag_2_S is linear with respect to time (Zou et al., 2013). Gen et al. (2011) found that in a high-humidity environment, creep corrosion occurred on the surface of samples comprising a copper base with a silver layer, and a large amount of whisker-like copper corrosion products appeared on the surface of the samples due to the presence of the layer. In addition, they found a large amount of microorganisms and corrosion products on the surface of PCBs that had been exposed in a clean atmospheric environment.

The authors have also investigated the influence of microbial colonization on PCBs exposed to natural environments. For example, *B. siamensis*, *B. cereus*, and *B. subtilis* have been isolated on PCBs exposed to the tropical rainforest climate in Xishuang Banna; the annual average humidity is 81%, the daytime temperature is generally above 30°C, and the content of S- and N-containing pollutants is very low. These environmental characteristics provide ideal conditions for the growth of microorganisms. In the present study, the corrosion behavior of a silver-plated copper PCB (PCB-ImAg) was investigated in the presence of *B. cereus*, which was isolated from exposure tests in Xishuang Banna. The exposure tests indicated that silver and copper ions released during the corrosion process of PCB-ImAg did not completely inhibit microbial growth. The tolerance of *B. cereus* to copper ions and silver ions was confirmed by comparing the growth curves of *B. cereus* in Luria-Bertani (LB) broth with different CuSO_4_ concentrations. The corrosion behavior of PCB-ImAg immersed in LB broth with and without *B. cereus* was investigated using electrochemical impedance spectroscopy (EIS), and the surface morphology was analyzed using confocal laser scanning microscopy (CLSM), field-emission scanning electron microscopy (FESEM), X-ray photoelectron spectroscopy (XPS), and Raman micro-spectroscopy.

## Materials and Methods

### Bacteria and Samples

The strain of *B. cereus* used for this study was isolated from PCB-ImAg after exposure at the Xishuang Banna test station for 3 months. The strain was cultured in LB agar (10 g/L tryptone, 5 g/L yeast extract, 10 g/L NaCl, and 15 g/L agar) at 30°C.

The samples used in the experiment were purchased from Shenzhen Sprint Circuits Co., Ltd. The thickness of the copper base material was 25 μm, and that of the silver layer was 0.38 μm. Prior to testing, the samples were degreased with ethanol before soaking in a 5% (v/v) glutaraldehyde solution for 5 h. Subsequently, the samples were transferred to a sterilized 75% ethanol solution and placed in a sterile operator station. The samples were used within 1 h after sterilization.

### Strain Culturing

The *B. cereus* strain was inoculated into a 150 mL Erlenmeyer flask containing 50 mL sterilized LB broth (10 g/L tryptone, 5 g/L yeast extract, and 10 g/L NaCl; the broth was autoclaved at 121°C for 25 min) at 30°C on a shaker (BSD-TX370, BOXUN, Shanghai) at a speed of 130 rpm for 24 h as a bacterial suspension. The cell concentration was determined using optical microscopy (Axio Lab.A1, Zeiss, Germany), and the concentration determined through hemocytometry was 12.9 × 10^10^/L.

### Growth Curve Measurements

#### Copper Ion Tolerance of *B. cereus*

A filtered (0.22 μm) solution of copper sulfate (CuSO_4_) was added to the LB broth to concentrations of 0, 100, 200, 300, and 400 mg/L.

#### Silver Ion Tolerance of *B. cereus*

A filtered (0.22 μm) solution of silver nitrate (AgNO_3_) was added to the broth (10 g/L tryptone, 5 g/L yeast extract, and 10 g/L NaNO_3_; the broth was autoclaved at 121°C for 25 min) to concentrations of 0, 25, and 50 mg/L.

The broth was poured into a 100-well plate, with each well containing 200 μL of broth and 2 μL of bacteria suspension. Three parallel samples were employed for each concentration. The growth curve at OD_600nm_ was monitored at 30°C for 15 days using an automatic growth curve analyzer (FP-1100-C, Bioscreen, Finland); measurements were collected every 2 h until the value was stable and then monitored every 12 h thereafter.

### Immersion Tests

The samples were inoculated in 150 mL flasks containing 50 mL sterilized LB broth with and without bacteria and cultured in an electric incubator (DHP–9052, YIHENG, Shanghai) at 30°C for a series of immersion times: 3, 6, and 15 days.

On each sampling day, the samples were removed from the broth with and without bacteria, immersed in PBS buffer for 3 min, placed in 2.5% (v/v) glutaraldehyde solution, and stored in the dark at 4°C for 12 h. The samples were rinsed with deionized water three times to remove the majority of the glutaraldehyde solution, dehydrated sequentially with 50, 70, 85, and 90 alcohol solutions (14 min each) and then dehydrated three times in 100% ethanol (15 min each).

### Surface Analyses

The surface morphology of the PCB-ImAg after immersion was determined by CLSM (VK-X250K, Keyence, Japan) and FESEM (QUANTA FEG 250, FEI, United States). The characteristics of the samples after immersion were measured using a focused ion beam (FIB, Zeiss, Germany). XPS (SigmaProbe, ThermoFisher, United States) and Raman micro-spectroscopy (CRS, inVia-Reflex, Renishaw, United Kingdom) were performed to determine the composition of the surface immersed in LB with and without *B. cereus* for 15 days. The XPS was equipped with an Al Kα monochromator and used a step size of 0.05 eV. The Raman spectra of the surface were recorded over 100–2000 cm^−1^ using laser excitation with a wavelength of 633 nm.

### EIS Measurements

EIS tests were conducted using a potentiostat (Reference 600, Camry Instruments Inc., United States) with a three-electrode system comprising a working electrode, a standard calomel electrode (SCE) as the reference electrode, and a platinum foil as the counter electrode. As the working electrodes, the samples were sealed with silicone rubber to create an exposed area of 1.2 cm^2^. EIS tests were performed at a stable-state open circuit potential (OCP), and the AC voltage was 10 mV at frequencies ranging from 0.01 to 10 kHz. The tests were conducted in the broth with and without *B. cereus* at 30°C for 15 days. The EIS results were fitted and analyzed with ZSimpWin software.

## Results

### Bacterial Growth

Over time, the surfaces of the samples of PCB-ImAg can diffuse copper and silver ions; thus, the tolerance of *B. cereus* to copper and silver ions can be obtained by observing the growth curve of the strain in broths containing the two types of ions.

**FIGURE 1:**
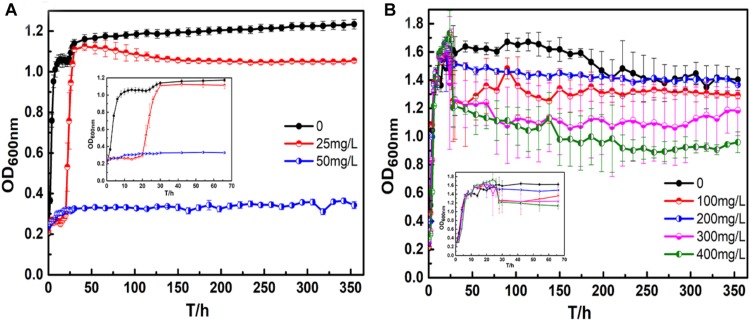
*Bacillus cereus* growth curves: **(A)** silver ions, and **(B)** copper ions.

Figure 1 shows the growth curve of *B. cereus* with silver and copper ions. For the growth curve of *B. cereus* with silver ions (Figure 1A), there were three growth phases for *B. cereus* in the broth containing 25 mg/L AgNO_3,_ namely, the adjustment, log, and stationary phases; concentrations of AgNO_3_ above 50 mg/L completely inhibited the growth of cells. Compared to the growth curve in the natural broth, the adjustment phase of *B. cereus* in the broth containing 25 mg/L AgNO_3_ was extended, and the optical density (OD) values were lower, which means that *B. cereus* has a certain silver ion tolerance: after a certain period of adaptation, the cells can grow and multiply, but toxic silver ions will affect the growth activity of the cells.

For the broth containing copper ions (Figure 1B), there were three growth phases for *B. cereus* in each broth: the lag, log, and stationary phases. In particular, there was no apparent decline until the end of the test because *B. cereus* can form spores to resist adverse conditions. The *B. cereus* in the CuSO_4_-containing broth exhibited a longer lag period than the *B. cereus* in the broth without CuSO_4_ to adapt to the new growing environment. After the log period, toxic metabolites accumulated in the broth, and the available nutrients were reduced, resulting in an imbalance in the number of living and dead cells. Thus, a short fluctuation appeared in the curves, and the bacterial growth entered the stable phase. In the broths with 100 and 200 mg/L CuSO_4_, the OD values of the bacterial suspension were almost the same as that of the control broth. However, in the broths with 300 and 400 mg/L CuSO_4_, the values were significantly lower. The adjustment period of bacterial growth became longer with increasing CuSO_4_ concentration, and the time to reach the stationary phase also increased. In the stationary phase, the OD values of the broths containing 100 and 200 mg/L CuSO_4_ were almost the same, whereas the values of the broths containing 300 and 400 mg/L CuSO_4_ were significantly lower.

### Surface Analysis

**FIGURE 2:**
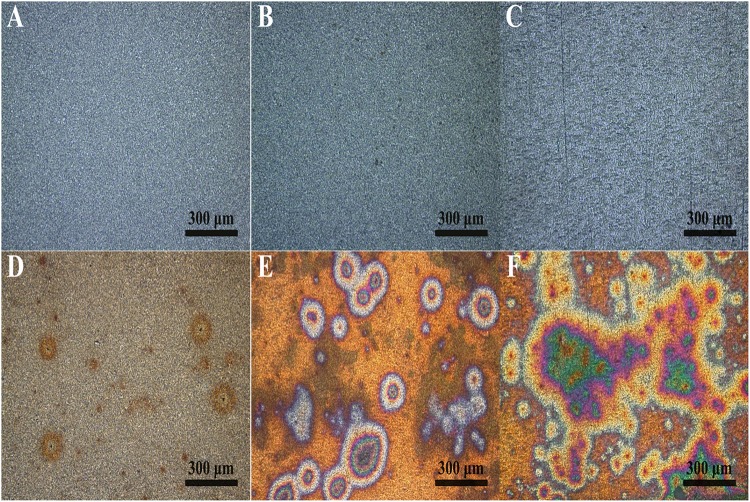
Macroscopic morphology determined using CLSM on the sample surface: **(A–C)** immersed in the broth without *B. cereus* for 3, 6, and 15 days and **(D–F)** immersed in the broth with *B. cereus* for 3, 6, and 15 days.

Figure 2 shows the macroscopic morphology of PCB-ImAg samples immersed in LB broth with and without *B. cereus* for 3, 6, and 15 days. In the sterile medium, the sample surface showed a slight color variation during the 15 days of immersion. However, the silver layer was intact. In comparison, the surface morphology of the samples immersed in the inoculated broth exhibited a rapid and significant change within the 15 days of culture. After 3 days of immersion (Figure 2D), although some corrosion spots could be observed on the PCB-ImAg surface, the color of the original silver layer was relatively clearly visible. Figure 2B shows that after 6 days, most of the surface of the sample showed a considerably increased corrosion circle; the center of the circle was black, indicating that the silver layer in the area had corroded. After 15 days of immersion (Figure 2C), the corrosion circle further developed into a large area, and the area inside the circle showed a green color, which indicated that the vast majority of copper corrosion products accumulated on the material surface. Most surface areas were yellowish brown, which means that the silver layer coating on the sample was incomplete.

**FIGURE 3:**
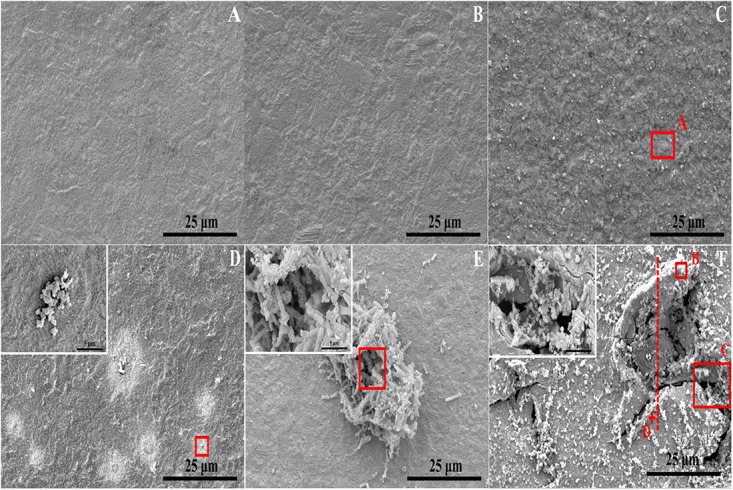
Microstructure determined via FESEM on sample surface: **(A–C)** immersed in the broth without *B. cereus* for 3, 6, and 15 days and **(D–F)** immersed in the broth with *B. cereus* for 3, 6, and 15 days.

The corrosion morphology of PCB-ImAg immersed in sterile and inoculated broths was further investigated by SEM (Figure 3). After immersion without bacteria for 3 and 6 days, the samples have relatively smooth and intact surfaces. Figure 3C shows that the surface was covered with some particles and became slightly rough after sample immersion in the sterile broth for 15 days. In the inoculated broth, the PCB-ImAg surface exhibited circular corrosion spots after 3 days of immersion (Figure 3D), which agreed with the optical images (Figure 2D). After the strains were cultured in the broth for 3 days, there was a certain concentration of metabolites in the broth, which accelerated microporous corrosion. Spores accumulated in the center of individual corrosion circles and nearby. During this period, the bacteria gathered into a cluster to adapt to the external environment. After immersion for 6 days (Figure 3E), a high concentration of cells had aggregated on the surface of the sample and formed a mature biofilm. The shape of the cells was rod-like, indicating that the cells were adapted to the test conditions and had developed from spores to vegetative cells. At same time, the bacteria in the biofilm possessed greater toxicity resistance (Gen et al., 2011). Therefore, the *B. cereus* cells began to rapidly propagate after the formation of the biofilm. Figure 3F shows the surface topography of a sample after being immersed in the inoculated broth for 15 days. The surface of the samples had severely deteriorated, and pits and peeling were observed. There was not only pitting on the surface but also large quantities of corrosion products that accumulated around the pits. A partial enlargement of area C shows spores and broken biofilm scattered on the surface of pits, which indicated that the cells had been attached here and formed a mature biofilm. After 15 days, there was a lack of nutrition, large amounts of toxic metabolites accumulated, and more copper and silver ions diffused in the broth, causing some cells to stop metabolizing and to form dormant spores.

**FIGURE 4:**
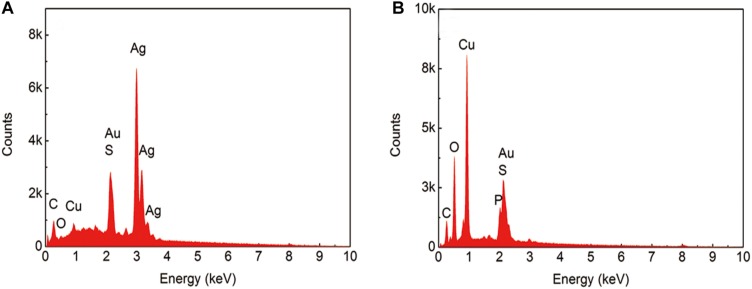
EDS results from the sample after immersion in the broth without **(A)** and with **(B)** *B. cereus* for 15 days.

Figure 4 shows the scanning results for areas A and B in Figure 3. In the sterile broth (Figure 4A), the silver content at the sample surface was much higher than the copper content, indicating that the silver layer remained intact and that the base copper was well protected. Figure 4B shows the energy dispersive X-ray spectroscopy (EDS) results for the corrosion pit on the sample immersed in the inoculated broth. The silver content in this area was much lower than the copper content, and the oxygen concentration was relatively high, indicating that the main component in the area may be copper oxide. The peak for phosphorous is also visible, which could be caused by the presence of the bacterial cells and their metabolites.

**FIGURE 5:**
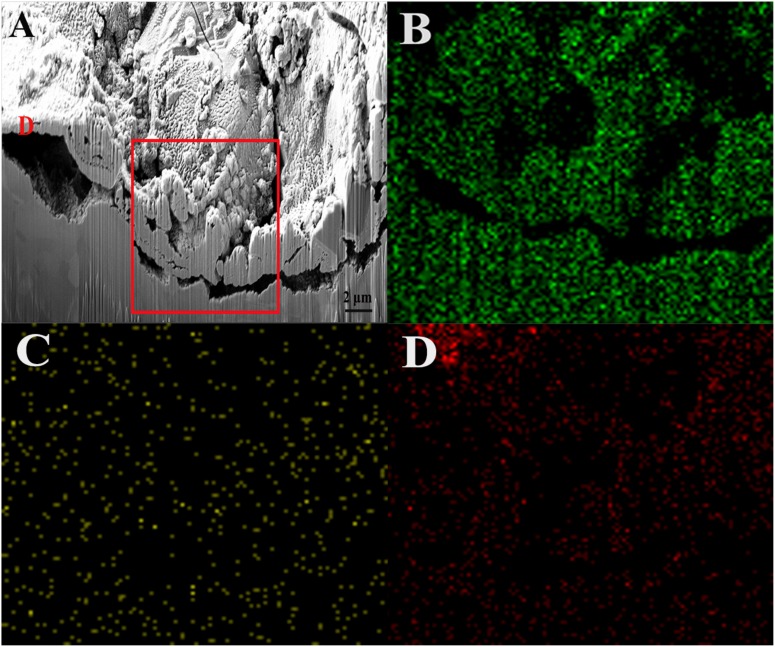
FIB image and element mapping of the damaged area of the sample after immersion in the broth with *B. cereus* for 15 days: **(A)** FIB, **(B)** Cu, **(C)** Ag, and **(D)** O.

Figure 5 shows a cross-sectional view of the dotted line shown in Figure 3F. As indicated, the value of Ag:Cu in area D was 16.64:26.21 according to the EDS result, which means that the silver layer partially remained on the sample surface. However, this view shows obvious pits and cracks under the surface, indicating a large loss of the base copper and silver, which led to the collapsed surface morphology. According to the results from elemental distribution mapping of the selected area, the Ag content was lower than the Cu content, indicating that the silver layer had almost completely dissolved in this area. The O content was slightly lower on the surface of defects than on the outside of pits (Figure 4B). Combined with the previous analysis, the adherence of the biofilm and elevated metabolism led to corrosion acceleration in areas where bacteria adhered.

**FIGURE 6:**
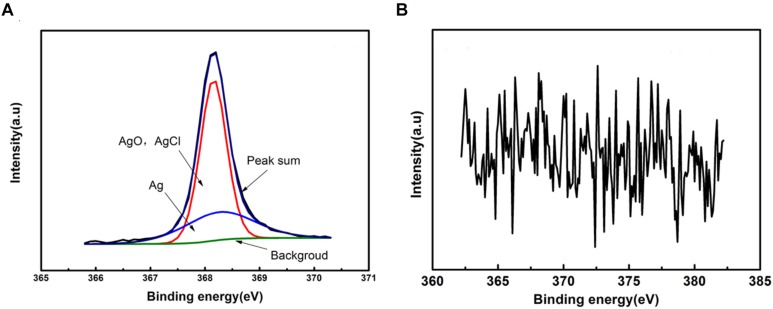
XPS spectra of the sample after immersed in the broth without **(A)** and with **(B)** *B. cereus* for 15 days.

Figure 6 shows the Ag 3d5/2 XPS spectra of the green area of PCB-ImAg immersed in broth with and without *B. cereus* for 15 days. The maximum point on the peaks represents the content of the corrosion products. For the sterile broth, the Ag 3d spectrum exhibits two peak components. Peak 1 appears at 368.16 eV and consists of pure silver (Ag), AgCl, and AgO (Qian et al., 2019). Peak 2, located at 368.26 eV, may be attributed to pure silver (Ag) (Hammond et al., 1975). This peak disappeared after 15 days of immersion in the inoculated broth, indicating that the silver layer was severely damaged.

**FIGURE 7:**
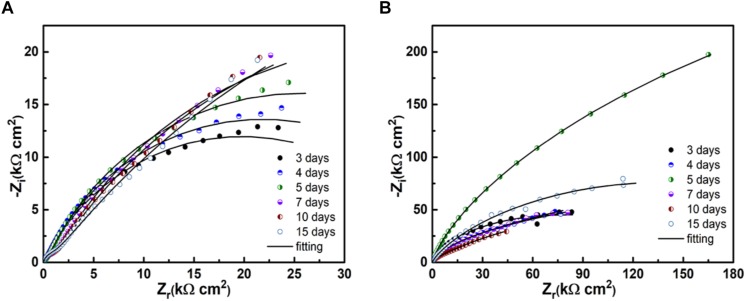
EIS of the samples after immersion in the broth without **(A)** and with **(B)** the *B. cereus.*

Figure 7 shows the Raman spectra obtained from the green area of samples after 15 days of immersion with and without *B. cereus*. The intensity of samples after immersion with *B. cereus* for 15 days was higher, indicating that there were more corrosion products in the presence of bacteria than in the control. In the lower wavenumber region, the samples exhibited the same main bands at 142, 525, 613, and 1356 cm^−1^. The bands at 525 and 625 cm^−1^ indicated the existence of Cu_2_O (Kosec et al., 2015). The bands at 142 cm^−1^ were assigned to the OCuO bending mode (Frost, 2003). The presence of CuO was validated by the broad doublet from 525 to 635 cm^−1^ (Chan et al., 1999; Xu et al., 1999). The band at 1356 cm^−1^ indicated the presence of Cu_2_Cl(OH)_3_ and CuCl_2_⋅2H_2_O (Frost, 2003). However, the band at 217 cm^−1^ was only observed in the control samples and was probably from Cu_2_O (Kosec et al., 2015). The band at 274 cm^−1^ was observed only in the samples after immersion with *B. cereus*. According to a previous study (Berger, 1976), this band could be assigned to copper sulfate pentahydrate (CuSO_4_⋅5H_2_O), which is formed by sulfur-containing metabolites. The Raman spectra indicated that the main corrosion products from the samples after immersion were Cu_2_O and CuO. However, owing to the effects of *B. cereus*, the products of the sample immersed in the broth with *B. cereus* also contained CuSO_4_⋅5H_2_O.

**FIGURE 8:**
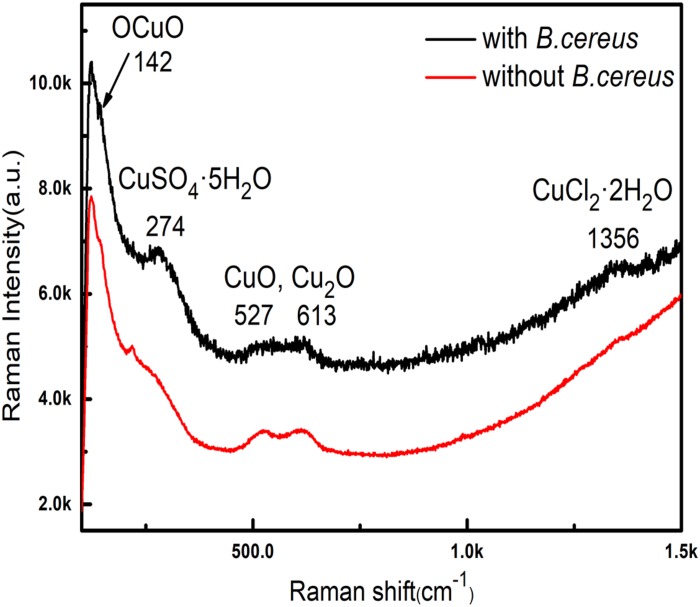
Raman spectra of samples after immersion in the broth with the *B. cereus* for 15 days.

Figure 8 shows the Nyquist diagrams of PCB-ImAg immersed in broth with and without *B. cereus* for different times. In both cases, the spectra have two time constants. In the presence of *B. cereus*, the size of the capacitance arc increased in the first 5 days of immersion and decreased after 7 days. The result of samples immersed with *B. cereus* showed that as the test time progressed, the formation of the biofilm and corrosion products followed a dynamic process.

**FIGURE 9:**
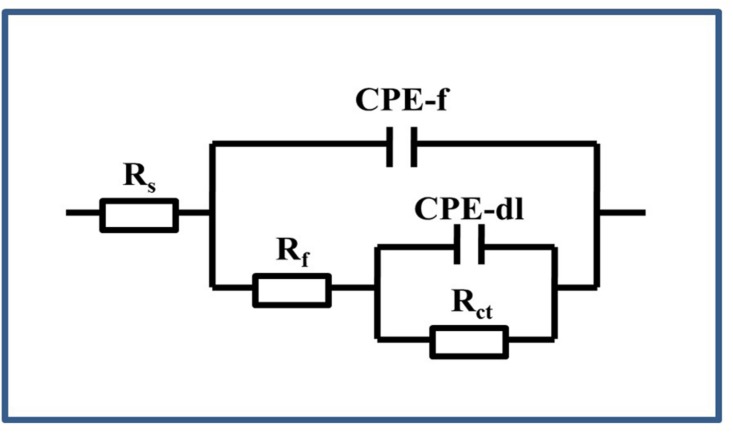
Equivalent circuit used for the EIS of the PCB-ImAg immersed in the broth with and without *B. cereus.*

Figure 9 shows the model used for the spectra after immersion with and without cells considering any defects on the surface. The related parameters are presented in Table 1. The variable *R*_*s*_ is the solution resistance; *R*_*f*_ is the resistance of the film; *R*_*ct*_ is the charge transfer resistance, representing the reaction rate of the base; and CPE-f and CPE-dl are parameters for the film and the double layer of the film electrolyte interface, respectively. A constant phase element (CPE) is often used instead of a capacitance to account for the non-ideal capacitance response due to the almost complete absence of pure capacitance in actual electrochemical processes (Mansfeld et al., 1993). Table 1 lists the corresponding parameters for both cases; the values for *R*_*s*_ are low because all of the broths used in the tests contained NaCl. For the sterile broth, the values of *R*_*ct*_ continued to increase during the test. Because microporous corrosion occurred slowly, corrosion products migrated through and blocked the micropores (Qian et al., 2018). For 15 days, some corrosion products covered the surfaces of the samples and accumulated on the surface, so that the film prevented the reaction of the base copper. *R*_*f*_ and *R*_*ct*_ for the samples immersed in *B. cereus* broth were much higher than those of the control sample, and the values first increased and then decreased. The variable *R*_*f*_ describes the film formation on the surface of the sample. This film formation process is complex, comprising the dynamic generation of biofilms and formation of corrosion products (Xu et al., 2018). Combined with the surface analysis shown in Figures 2–5, we can infer that on the third day, the *B. cereus* accumulated on the surface increased the value of *R*_*f*_. After 5 days, the bacteria had grown to form a mature biofilm adhering to the surface and preventing corrosion of the base copper. As the biofilm on the surface aged and broke, *R*_*ct*_ and *R*_*f*_ decreased sharply. On the fifteenth day, there were obvious defects on the sample surface. The FIB image shows that the base copper at the silver rupture was directly exposed to the solution, which increased the corrosion rate of the base copper, and corrosion products accumulated on the surface preventing reaction of the base material.

**TABLE 1:** Fitting parameters of EIS of PCB-ImAg after immersed in the broth.

**T/days**	***R*_*s*_ (Ω cm^2^)**	**CPE-f (μF cm^−2^)**	***R*_*f*_ (kΩ cm^2^)**	**CPE-dl (μF cm^−2^)**	***R*_*ct*_ (kΩ cm^2^)**
**Without *B. cereus***					
3	8.2	27.0	0.3	94.0	39.8
4	7.8	22.9	0.3	109.0	43.6
5	7.6	19.5	0.4	123.2	50.9
7	7.0	16.6	1.2	145.6	70.2
10	7.1	16.1	1.3	148.0	81.9
15	7.1	15.3	1.3	140.1	119.1
**With *B. cereus***					
3	10.2	26.2	2.5	15.8	207.6
4	9.1	14.5	4.6	24.1	369.5
5	9.2	22.1	46.8	8.4	1163.0
7	8.2	14.9	6.0	26.9	446.6
10	8.6	20.2	4.6	59.7	217.9
15	9.5	12.6	6.9	17.3	294.2

## Discussion

The growth test showed that the strains used in the test have a certain tolerance to silver and copper ions. The bacteria could grow and reproduce in broth containing 25 mg/L AgNO_3_, and CuSO_4_ concentrations below 200 mg/L did not obviously affect cell growth in the growth test. This growth characteristic greatly assisted the bacteria to adhere and grow on the surface of PCB-ImAg. It is known that silver ions have strong antibacterial properties (Teitzel and Parsek, 2003). The trace element copper is an essential nutrient in a certain concentration (Santo et al., 2011); however, when that concentration is exceeded, the ions reduce the microbial activity and number. Letelier et al. (2010) reported that the non-specific protein-binding properties of copper ions can increase their pro-oxidant activity when damaging biomolecules. However, a low concentration of copper ions can also increase enzyme activity and protein synthesis, causing cells to have more tightly packed arrays intrinsic to plasma membranes (Prior and Dalton, 1985). Therefore, there is a difference in the tolerance of the bacteria to the two ions. Furthermore, the complexation of metal ions by the organics in solution (Thurman et al., 1989) and the biosorption of heavy metal ions onto *B. cereus* biomass has previously been reported (Hasan et al., 2016); these effects reduced toxicity due to a decrease in the effective ion concentration in the solution. For example, we have found that when 400 mg/L CuSO_4_ was added, the concentration of copper ion in LB broth was only 150 mg/L by ICP. Despite this significant reduction in free copper ions, the *B. cereus* used in this experiment has relatively high copper tolerance compared with the strains reported in other studies. According to Sokhn et al. (2001) and Teitzel and Parsek (2003), 770 ppm copper ions could significantly reduce microbial activity of soil bacteria, and the growth of *Pseudomonas aeruginosa* was completely inhibited in the broth with 128 ppm copper ions. In an earlier work, Singh et al. (1985) also reported a 98% decrease in the cell viability of Yersinia enterocolitica after 2 days in the presence of 1 mg/L copper.

Surface analysis of the samples indicated that the corrosion behavior of PCB-ImAg with *B. cereus* is related to the biofilm formation and metabolism. According to a previous study (Zou et al., 2013), PCB-ImAg has good protection against Cl^–^; thus, in the sterile broth, only slight corrosion occurred on the surface of the material after immersion for 15 days, and the silver layer was intact, protecting the base copper. The silver layer was very thin, and there were defects, such as micropores, on the surface of the sample. Therefore, the copper base was exposed to the broth, which led to microporous corrosion. As shown in Figure 3D, the areas of microporous corrosion were weak points that had more copper corrosion products, a thinner silver layer, and less toxicity and favored further growth of the strain. When the strains formed mature biofilms, the large amount of oxygen consumed by cellular respiration led to different oxygen concentrations inside and outside of the biofilm, causing oxygen concentration corrosion. Additionally, the large amount of metabolites accelerated microporous corrosion and creep corrosion. This result was confirmed by the differences in O content in distinct areas, as shown in the EDS results.

The results of XPS and Raman analyses show different compositions of the compounds in both cases. In the bacterial broth, the corrosion products contained copper oxides and chlorides; CuSO_4_⋅5H_2_O was present on the surface of the sample. In addition, elemental Ag was not clearly apparent in the peaks (Figure 6B), indicating that the silver layer in the area had been damaged.

Biofilm formation and metabolic activity are closely related according to EIS results. In the sterile broth, as the immersion time increased, the corrosion process of samples was slow and a dense silver layer protected the base copper by hindering further corrosion of the base. Moreover, corrosion products that blocked micropores on the surface could also retard copper base reaction. The changes in the EIS results for the bacterial broth were caused by interactions between the PCB-ImAg and bacterial metabolism. At the beginning of the test, microporous corrosion was accelerated by bacteria, and corrosion products contained dense Cu_2_O (Figure 7; Strandberg, 1998), which caused the values of R_*ct*_ to be higher than that of control at the same time. With the adsorption of the mature biofilm, corresponding sharp increases were observed in R_*f*_ and R_*ct*_. In addition, high concentration of metabolites under the biofilm continued to corrode the copper base (Xu et al., 2017). After the biofilm broke down, the copper base exposure to the broth aggravated galvanic corrosion and caused further consumption of the copper base. Moreover, the thick film of copper corrosion products covered on the surface increased R_*ct*_ slightly.

PCB-ImAg is usually used in atmospheric environment, the composition of which is quite complex and variable and may include H_2_S, SO_2_, HCl, particulates, etc. To study atmospheric corrosion, simplified solutions were typically used simulating only the ionic compositions and concentrations. These solutions, however, are not suitable to study the MIC of PCB due to the lack of sufficient organic nutrients to support the bacterial growth. Under such considerations, LB broth was used as test medium in this study to allow bacteria to grow and reproduce, so the corrosion acceleration of the PCB-ImAg also be amplified and the authors could observe the interaction between the materials and the bacteria could be more clearly investigated. The results of the sterile control indicated the organic ingredients in LB broth did not present significant corrosiveness to the PCB as only slight corrosion had occurred on the PCB surface. Therefore, the rapid deterioration of the PCB surface in *B. cereus* inoculated medium was closely related to the life activities of the bacteria.

Nevertheless, a noteworthy limitation in this study was the use of much more concentrated organic nutrients than the actual atmospheric environment may contain. In this sense, future works will explore the uses of culture media that can more closely simulate the atmospheric environments. For example, a more realistic culture medium could be obtained by analyzing the composition of the thin electrolyte film formed bacteria-grown specimens exposed under real atmosphere. The simulated solution may also be supplemented with varied concentration of organic nutrients to further understand their roles in the MIC of PCBs. Interestingly, previous studies (Qian et al., 2018; Gu et al., 2019) have found that microbes starved with organic nutrients may more aggressively corrode metals via extracellular electron transfer using metals as electron donors.

## Conclusion

The results from all the tests showed that the PCB-ImAg samples were unable to resist attacks from *B. cereus* in an aerobic medium. The following conclusions can be drawn from the results:

(1) *B. cereus* has a high copper tolerance and a certain silver tolerance, as 400 mg/L of copper sulfate cannot completely inhibit its growth.
(2) In the LB broth, MIC caused by *B. cereus* was the result of several factors. The biofilm of *B. cereus* promotes microporous corrosion possibly by producing metabolites. Simultaneously, the biofilm that adhered to the surface probably led to an oxygen concentration cell, and metabolites accelerated the microporous corrosion.
(3) The EIS data show that *B. cereus* promotes corrosion of the base copper, leading to a very thick layer of corrosion products accumulating on the surface. This result was demonstrated in the spectra, in which all the *R*_*f*_ and *R*_*ct*_ values were higher than those in the sterile broth.

## Data Availability

The datasets generated for this study are available on request to the corresponding author.

## Author Contributions

YH and KX designed the experiments. YH carried out the experiments, analyzed the data, and drafted the manuscript. PY and RX assisted in carrying out some experiments. DZ, CD, and XL revised the manuscript. All authors read and approved the final manuscript.

## Conflict of Interest Statement

The authors declare that the research was conducted in the absence of any commercial or financial relationships that could be construed as a potential conflict of interest.

## References

[B1] Berger J. (1976). Infrared and Raman spectra of CuSO4, 5H2O; CuSO4, 5D2O; and CuSeO4, 5H2O. *J. Raman Spectrosc.* 5 103–114. 10.1002/jrs.1250050202

[B2] Chan H. Y. H. Takoudis C. G. Weaver M. J. (1999). Oxide film formation and oxygen adsorption on copper in aqueous media as probed by surface-enhanced Raman spectroscopy. *J. Phys. Chem. B.* 103 357–365. 10.1021/jp983787c

[B3] Flemming H. C. (1996). “Biofuling and microbilogically influenced corrosion (MIC): an economical and technical overview,” in *Microbial Deterioration Materiaols*, eds Heitz E. Flemming H.-C. Sand W. (Heidelberg: Springer), 5–14. 10.1007/978-3-642-80017-7_2

[B4] Frost R. L. (2003). Raman spectroscopy of selected copper minerals of significance in corrosion. *Spectrochim. Acta Part A* 59 1195–1204. 10.1016/s1386-1425(02)00315-3 12659888

[B5] Gen W. Chen X. Hu A. Li M. (2011). Effect of Ag on oxidation of Cu-base leadframe. *Microelectron. Reliab.* 1 866–870. 10.1016/j.microrel.2010.10.010

[B6] Gu T. Jia R. Unsal T. Xu D. (2019). Toward a better understanding of microbiologically influenced corrosion caused by sulfate reducing bacteria. *J. Mater. Sci. Technol.* 35 631–636. 10.1016/j.jmst.2018.10.026

[B7] Hammond J. S. Gaarenstroom S. W. Winograd N. (1975). X-ray photoelectron spectroscopic studies of cadmium-and silver-oxygen surfaces. *Anal. Chem.* 47 2193–2199. 10.1021/ac60363a019

[B8] Hasan H. A. Abdullah S. R. S. Kofli N. T. Yeoh S. J. (2016). Interaction of environmental factors on simultaneous biosorption of lead and manganese ions by locally isolated *Bacillus cereus*. *J. Ind. Eng. Chem.* 37 295–305. 10.1016/j.jiec.2016.03.038

[B9] Hou B. Li X. Ma X. Du C. Zhang D. Zheng M. (2017). The cost of corrosion in China. *npj Mater. Degrad.* 1:4.

[B10] Huang H. Dong Z. Chen Z. Guo X. (2011). The effects of Cl- ion concentration and relative humidity on atmospheric corrosion behaviour of PCB-Cu under adsorbed thin electrolyte layer. *Corros. Sci.* 53 1230–1236. 10.1016/j.corsci.2010.12.018

[B11] Huang L. Li Z. Lou Y. Cao F. Zhang D. Li X. (2018). Recent advances in scanning electrochemical microscopy for biological applications. *Materials* 11:1389. 10.3390/ma11081389 30096895 PMC6119995

[B12] Jia R. Unsal T. Xu D. Lekbach Y. Gu T. (2019). Microbiologically influenced corrosion and current mitigation strategies: a state of the art review. *Int. Biodeterior. Biodegrad.* 137 42–58. 10.1016/j.ibiod.2018.11.007

[B13] Juzeliūnas E. Ramanauskas R. Lugauskas A. Samulevičienė M. Leinartas K. (2005). Microbially influenced corrosion acceleration and inhibition. EIS study of Zn and Al subjected for two years to influence of Penicillium frequentans, *Aspergillus niger* and *Bacillus mycoides*. *Electrochem. Commun.* 7 305–311. 10.1016/j.elecom.2005.01.012

[B14] Kosec T. Qin Z. Chen J. Legat A. Shoesmith D. W. (2015). Copper corrosion in bentonite/saline groundwater solution: effects of solution and bentonite chemistry. *Corros. Sci.* 90 248–258. 10.1016/j.corsci.2014.10.017

[B15] Letelier M. E. Sánchez-Jofré S. Peredo-Silva L. Cortés-Troncoso J. Aracena-Parks P. (2010). Mechanisms underlying iron and copper ions toxicity in biological systems: pro-oxidant activity and protein-binding effects. *Chem. Biol. Interact.* 188 220–227. 10.1016/j.cbi.2010.06.013 20603110

[B16] Li Y. Xu D. Chen C. Li X. Jia R. Zhang D. (2018). Anaerobic microbiologically influenced corrosion mechanisms interpreted using bioenergetics and bioelectrochemistry: a review. *J. Mater. Sci. Technol.* 34 1713–1718. 10.1016/j.jmst.2018.02.023

[B17] Mansfeld F. Wang Y. Lin S. H. Xiao H. Shih H. (1993). “Detection and monitoring of localized corrosion by EIS,” in *Electrochemical Impedance: Analysis and Interpretation.*, eds Scully J. Silverman D. Kendig M. West Conshohocken, PA: ASTM International 297–312. 10.1520/stp18076s

[B18] Nakamura K. Silver S. (1994). Molecular analysis of mercury-resistant *Bacillus* isolates from sediment of Minamata Bay, Japan. *Appl. Environ. Microbiol.* 60 4596–4599. 7811095 10.1128/aem.60.12.4596-4599.1994 PMC202026

[B19] Nourbakhsh M. N. Kiliçarslan S. Ilhan S. Ozdag H. (2002). Biosorption of Cr6+, Pb2+ and Cu2+ ions in industrial waste water on *Bacillus* sp. *Chem. Eng. J.* 85 351–355. 10.1016/s1385-8947(01)00227-3

[B20] Novikova N. De Boever P. Poddubko S. Deshevaya E. Polikarpov N. Rakova N. (2006). Survey of environmental biocontamination on board the International Space Station. *Res. Microbiol.* 157 5–12. 10.1016/j.resmic.2005.07.010 16364606

[B21] Prior S. D. Dalton H. (1985). The effect of copper ions on membrane content and methane monooxygenase activity in methanol-grown cells of *Methylococcus capsulatus* (Bath). *Microbiology* 131 155–163. 10.1099/00221287-131-1-155

[B22] Qian H. Yang J. Lou Y. ur Rahman O. Li Z. Ding X. (2019). Mussel-inspired superhydrophilic surface with enhanced antimicrobial properties under immersed and atmospheric conditions. *Appl. Surf. Sci.* 465 267–278. 10.1016/j.apsusc.2018.09.173

[B23] Qian H. Zhang D. Lou Y. Li Z. Xu D. Du C. (2018). Laboratory investigation of microbiologically influenced corrosion of Q235 carbon steel by halophilic archaea *Natronorubrum tibetense*. *Corros. Sci.* 145 151–161. 10.1016/j.corsci.2018.09.020

[B24] Qu Q. He Y. Wang L. Xu H. Li L. Chen Y. (2015). Corrosion behavior of cold rolled steel in artificial seawater in the presence of *Bacillus subtilis* C2. *Corros. Sci.* 91 321–329. 10.1016/j.corsci.2014.11.032

[B25] Russo S. G. Henderson M. J. Hinton B. R. W. (2002). Corrosion of an aircraft radar antenna waveguide. *Eng. Fail. Anal.* 9 423–434. 10.1016/S1350-6307(01)00028-0

[B26] Santo C. E. Lam E. W. Elowsky C. G. Quaranta D. Domaille D. W. Chang C. J. (2011). Bacterial killing by dry metallic copper surfaces. *Appl. Environ. Microbiol.* 77 794–802. 10.1128/aem.01599-10 21148701 PMC3028699

[B27] Shivaji S. Chaturvedi P. Suresh K. Reddy G. S. N. Dutt C. B. S. Wainwright M. (2006). *Bacillus aerius* sp. nov., *Bacillus aerophilus* sp. nov., *Bacillus stratosphericus* sp. nov. and *Bacillus altitudinis* sp. nov., isolated from cryogenic tubes used for collecting air samples from high altitudes. Int. J. Syst. Evol. Microbiol. 56 1465–1473. 10.1099/ijs.0.64029-0 16825614

[B28] Siddiqui S. Siddiqui Z. A. Ahmad I. (2005). Evaluation of fluorescent *Pseudomonads* and *Bacillus*, isolates for the biocontrol of a wilt disease complex of pigeonpea. *World J. Microbiol. Biotechnol.* 21 729–732. 10.1007/s11274-004-4799-z

[B29] Singh A. LeChevallier M. W. McFeters G. A. (1985). Reduced virulence of Yersinia enterocolitica by copper-induced injury. *Appl. Environ. Microbiol.* 50 406–411. 4051485 10.1128/aem.50.2.406-411.1985 PMC238634

[B30] Sokhn J. De Leij F. A. A. M. Hart T. D. Lynch J. M. (2001). Effect of copper on the degradation of phenanthrene by soil micro-organisms. *Lett. Appl. Microbiol.* 33 164–168. 10.1046/j.1472-765x.2001.00972.x 11472527

[B31] Strandberg H. (1998). Reactions of copper patina compounds—II. Influence of sodium chloride in the presence of some air pollutants. *Atmos. Environ.* 32 3521–3526. 10.1016/S1352-2310(98)00058-2

[B32] Teitzel G. M. Parsek M. R. (2003). Heavy metal resistance of biofilm and planktonic *Pseudomonas aeruginosa*. *Appl. Environ. Microbiol.* 69 2313–2320. 10.1128/aem.69.4.2313-2320.2003 12676715 PMC154819

[B33] Thurman R. B. Gerba C. P. Bitton G. (1989). The molecular mechanisms of copper and silver ion disinfection of bacteria and viruses. *Crit. Rev. Environ. Sci. Technol.* 18 295–315. 10.1080/10643388909388351

[B34] Xiao K. Yi P. Yan L. Bai Z. Dong C. Dong P. (2017). Corrosion behavior of silver-plated circuit boards in a simulated marine environment with industrial pollution. *Materials* 10: 762. 10.3390/ma10070762 28773121 PMC5551805

[B35] Xu D. Gu T. (2014). Carbon source starvation triggered more aggressive corrosion against carbon steel by the *Desulfovibrio vulgaris* biofilm. *Int. Biodeterior. Biodegrad.* 91 74–81. 10.1016/j.ibiod.2014.03.014

[B36] Xu D. Li Y. Gu T. (2016). Mechanistic modeling of biocorrosion caused by biofilms of sulfate reducing bacteria and acid producing bacteria. *Bioelectrochemistry* 110 52–58. 10.1016/j.bioelechem.2016.03.003 27071053

[B37] Xu D. Li Y. Song F. Gu T. (2013). Laboratory investigation of micro-biologically influenced corrosion of C1018 carbon steel by nitrate reducing bacterium *Bacillus licheniformis*. *Corros. Sci.* 77 385–390. 10.1016/j.corsci.2013.07.044

[B38] Xu D. Xia J. Zhou E. Zhang D. Li H. Yang C. (2017). Accelerated corrosion of 2205 duplex stainless steel caused by marine aerobic *Pseudomonas aeruginosa* biofilm. *Bioelectrochemistry* 113 1–8. 10.1016/j.bioelechem.2016.08.001 27578208

[B39] Xu D. Zhou E. Zhao Y. Li H. Liu Z. Zhang D. (2018). Enhanced resistance of 2205 Cu-bearing duplex stainless steel towards microbiologically influenced corrosion by marine aerobic *Pseudomonas aeruginosa* biofilms. *J. Mater. Sci. Technol.* 34 1325–1336. 10.1016/j.jmst.2017.11.025

[B40] Xu J. F. Ji W. Shen Z. X. Li W. S. Tang S. H. Ye X. R. (1999). Raman spectra of CuO nanocrystals. *J. Raman Spectrosc.* 30 413–415. 10.1002/(SICI)1097-4555(199905)30:5<413::AID-JRS387<3.0.CO;2-N

[B41] Yan L. Xiao K. Yi P. Dong C. Wu J. Bai Z. (2017). The corrosion behavior of PCB-ImAg in industry polluted marine atmosphere environment. *Mater. Des.* 115 404–414. 10.1016/j.matdes.2016.11.074

[B42] Yan L. Xiao K. Yi P. Dong C. Wu J. Mao C. (2016). Surface analysis of silver-plated circuit boards in a salt-spray environment. *J. Alloys Compd.* 688 301–312. 10.1016/j.jallcom.2016.07.207

[B43] Yi P. Xiao K. Ding K. Yan L. Dong C. Li X. (2016). Initial corrosion behavior of a copper-clad plate in typical outdoor atmospheric environments. *Electron. Mater. Lett.* 12 163–170. 10.1007/s13391-015-5309-1

[B44] Yousaf M. Ali I. Arif M. A. Mustafa G. M. Ahmad S. Afzal N. (2015). Effects of microbiologically influenced corrosion by *Bacillus megaterium* bacteria on the mechanical properties of Al-Cu Alloy. *Mater. Today Proc.* 2 5669–5673. 10.1016/j.matpr.2015.11.107

[B45] Zou S. Li X. Dong C. Ding K. Xiao K. (2013). Electrochemical migration, whisker formation, and corrosion behavior of printed circuit board under wet H2S environment. *Electrochim. Acta* 114 363–371. 10.1016/j.electacta.2013.10.051

